# Supplementary material for: Intragenic transcription from defective HIV proviruses triggers interferon responses in myeloid cells
Source: J Virol. 2026 Aug 10;100(9):e00552-26. doi: 10.1128/jvi.00552-26 (PMC13595952; doi:10.1128/jvi.00552-26)
Supplement: Supplementary file 1 [file jvi.00552-26-s0007.pdf]

SUPPLEMENTAL FIGURE LEGENDS

Supplemental Figure 1: **Confirmation of knockdowns by PCR.** White arrows indicate PCR amplicons arising from CRISPR-modified HIV-1 proviruses. A-C) PCR was used to amplify genomic DNA as shown in Fig 2B from Jurkat-Cas9 cells infected with NL4-3 and transfected with either scrambled or 5'deleting gRNA-expressing plasmids. Each panel represents a different replicate within Jurkat-Cas9 cells. D-F) PCR products from genomic DNA isolated from MDMs harboring full length or modified HIV-1 provirus. Each gel represents a unique MDM donor with 3 replicates per infected condition per donor. G-I) PCR products from genomic DNA isolated from PMA-differentiated THP1s (G), PMA-differentiated MAVS-knockdown THP1s (H), PMA-differentiated MDA5-knockdown THP1s (I), PMA-differentiated RIG-I knockdown THP1s, and (J) PMA-differentiated cGAS knockdown THP1s. Each gel represents three replicates for scrambled infected controls and three replicates for 5'deleted provirus for each cell line.

Supplemental Figure 2: **Confirmation of modified HIV-1 proviruses by sequencing.** PCR was run on genomic DNA from infected cells to measure CRISPR-Cas9 modification. PCR amplicons were sequenced by nanopore sequencing. The number of reads mapping to either intact proviruses (1.5kb) or CRISPR-modified proviruses (400bp) were used to quantify the percent of modification. Data are shown as percent of total reads per sample. A) PCR amplicon sequencing from Jurkat infections. Data are shown as an average of two replicates. B) PCR amplicon sequences from MDMs. Data are shown as an average of three infections per donor. Donor numbers are matched between scrambled control and

5’modified provirus conditions. C) PCR amplicon sequencing from THP1 infections. Data is shown as an average of three replicates.

Supplemental Figure 3 CRISPR-Cas9 RNP treatment did not impact HIV-1 infection. qPCR was used to quantify copies of *env* DNA per ng input genomic DNA to determine the overall copies of provirus following CRISPR modification. Titration of NL-43 was used determine *env* copy. Data are from MDMs (A), PMA-differentiated THP1s (B), PMA-differentiated MAVS-knockdown THP1s (C), and PMA-differentiated MDA5-knockdown THP1s (D).

Supplemental Figure 4: **Targeting *hprt* in MDMs does not induce interferons.** A) qPCR confirming knockdown of endogenous human gene *hprt* from genomic DNA in MDMs. *hprt*-targeting CRISPR-Cas9 RNPs were formed as described in the methods. DNA was harvested 48 hours post transfection. B) Supernatant from modified MDMs was added to HEK-293-ISRE-Luc cells and compared to the addition of DMEM (negative control) and recombinant Interferon alpha (positive control) to the reporter cell line.

Supplemental Figure 5: Defective HIV-1 RNAs are capped and poly-A tailed. RNA was isolated from PMA-differentiated THP1 cells (A-B) or MDMs (C-D). A portion of this RNA was bound to eIF4e-GFP coated beads which were used to capture 5’capped mRNA. Levels of UTR-containing and UTR-deficient transcripts were determined using multiplex RTddPCR. Each panel reflects an individual replicate for THP1s or MDMs. Each replicate in MDMs was performed on a unique donor.

Supplemental Figure 6: 5’ capped RPL13a mRNA. A-B) RTqPCR for RPL13a, a 5’capped mRNA transcript constitutively expressed in all cells, on RNA from MDMs (A) or PMA-differentiated THP1s (B) before selection for 5’cap mRNA or after selection for 5’cap mRNA. Each data point represents a biological replicate for a total of three replicates.
