# Supplementary material for: Intragenic transcription from defective HIV proviruses triggers interferon responses in myeloid cells
Source: J Virol. 2026 Aug 10;100(9):e00552-26. doi: 10.1128/jvi.00552-26 (PMC13595952; doi:10.1128/jvi.00552-26)
Supplement: Supplementary file 2 [file jvi.00552-26-s0008.pdf]

SUPPLEMENTAL TABLES

Supplemental Table 1: Primer and Probe Sequences

Target	Forward Sequence (5'-3')	Reverse Sequence (5'-3')
IP10	CGAAGGCCATCAAGAATTTACTG	CCATCCTTGGAAGCACTG
RPL13a	CAAGCGGATGAACACCAAC	CGCTTTTTCTTGTCGTAGGG
TNFα	TCATGGGTTTCTCCACCAAG	GTGCCAACAACTGCCTTTATATG
IL-8	CCACCGGAAGGAACCATCTC	TTCCTTGGGGTCCAGACAGA
IL-6	CCTGAGAAAGGAGACATGTAA	CACCAGGCAAGTCTCCTCATTG
IFN2α	TGGGCTGTGATCTGCCTCAAAC	CAGCCTTTTGGAACTGGTTGCC
IFNγ	GAGTGTGGAGACCATCAAGGAAG	TGCTTTGCGTTGGACATTCAAGTC
Leu-tRNA	ACACCCTCCTAGCCTTACTAC	GATATAGGGTCGAAGCCGC
MALAT1	TGGTGGGGTGGGTTTAGGTA	TTCCCCCAGCTTCCCAATTC
IRF1	CTCTGAAGCTACAACAGATGAG	GTAGACTCAGCCCAATATCCC
Psi (for ddPCR)	AGTGGTGCAGAGAGAAAAAAGAGC	GTCTGGCCTGTACCGTCAGC
Long LTR (for ddPCR)	GCCTCAATAAAGCTTGCCTTGA	GGGCGCCACTGCTAGAGA
Env (for ddPCR)	AGTGGTGCAGAGAGAAAAAAGAGC	GTCTGGCCTGTACCGTCAGC
Nef (for ddPCR)	GGTGGGAGCAGTATCTCGAGA	TGTAAGTCATTGGTCTTAAAGGTACCTGAGG
LTR gRNA plasmid cloning primers	CACCGTTGAACCAGAGCAAGTAGAA	AAACTTCTACTTGCTCTGGTTCAAC
Gag gRNA plasmid cloning primers	CACCGGATAGATGTAAAAGACACCA	AAACTGGTGTCTTTTACATCTATCC
Scrambled gRNA plasmid cloning primers	CACCGCACTACCAGAGCTAACTCA	AAACTGAGTTAGCTCTGGTAGTGC
Primers to confirm CRISPR deletion	CAAGAGATCCTTGATCTGTGG	GTTCCTGCTATGTCACTTC
**Probes**	**Fluorophore**	**Sequence (5'-3')**
Long LTR	FAM	CCAGAGTCACACAACAGACGGGCACA
Env	Vic	CCTTGGGTTCTTGGGA
Nef	FAM	CCAGGCACAAGCAGCATT

Supplemental Table 2: gRNA Sequences

gRNA for RNPs	Sequence
LTR	GGAUUGGCAGAACUACACACCGUUUUAGAGCUAGAAAUAGCAAGUUAAAAUAAGG CUAGUCCGUUAUCAACUUGAAAAAGUGGCACCGAGUCGGUGCUUUUUUUU
Gag	GGGAUAGAUGUAAAAGACACCAGUUUUAGAGCUAGAAAUAGCAAGUUAAAAUAAG GCUAGUCCGUUAUCAACUUGAAAAAGUGGCACCGAGUCGGUGCUUUUUUUU
Scrambled	GGCACUACCAGAGCUAACUCAGUUUUAGAGCUAGAAAUAGCAAGUUAAAAUAAGG CUAGUCCGUUAUCAACUUGAAAAAGUGGCACCGAGUCGGUGCUUUUUUUU

Supplemental Table 3: Concentration of RNP components in master mix

Cell Type	HEK293Ts	PMA-differentiated THP1s	Primary MDMs
Concentration gRNA	320-640nM	200nM	800nM
Concentration cas9	160nM	200nM	400nM
Concentration lipofectamine	30nL/uL	30nL/uL	30nL/uL
Concentration Cas9 PLUS Enhancer	24nL/uL	50nL/uL	50nL/uL
Inoculum volume (12-well plate)	100uL	100uL	100uL

Supplemental Table 4: Test for normal distribution	UTR+RNA	UTR-RNA	Total RNA	IL-6	TNF-a	IP-10
**D'Agostino & Pearson test**						
**K2**	0.2507	16.92	3.339	0.848	4.971	3.045
**P value**	0.8822	0.0002	0.1884	0.6544	0.0833	0.2182
**Passed normality test (alpha=0.05)?**	Yes	No	Yes	Yes	Yes	Yes
**P value summary**	ns	***	ns	ns	ns	ns
**Anderson-Darling test**						
**A2***	0.4156	1.137	0.2943	0.1861	1.771	0.4851
**P value**	0.3052	0.0044	0.5668	0.8941	0.0001	0.2042
**Passed normality test (alpha=0.05)?**	Yes	No	Yes	Yes	No	Yes
**P value summary**	ns	**	ns	ns	***	ns
**Shapiro-Wilk test**						
**W**	0.9504	0.8132	0.9603	0.9857	0.8313	0.9196
**P value**	0.3221	0.0008	0.4945	0.9806	0.0016	0.0745
**Passed normality test (alpha=0.05)?**	Yes	No	Yes	Yes	No	Yes
**P value summary**	ns	***	ns	ns	**	ns
**Kolmogorov-Smirnov test**						
**KS distance**	0.1281	0.1961	0.1165	0.1089	0.2619	0.1238
**P value**	>0.1000	0.0274	>0.1000	>0.1000	0.0004	>0.1000
**Passed normality test (alpha=0.05)?**	Yes	No	Yes	Yes	No	Yes
**P value summary**	ns	*	ns	ns	***	ns
Number of values	22	22	22	22	22	22

Supplemental Table 4 (cont.): Test for normal distribution	IL-8	IL-12p70	Age @ enrollment	ART Years	Absolute CD4 count (cell/mm3)
**D'Agostino & Pearson test**					
**K2**	5.18	7.707	9.806	1.72	5.433
**P value**	0.075	0.0212	0.0074	0.4232	0.0661
**Passed normality test (alpha=0.05)?**	Yes	No	No	Yes	Yes
**P value summary**	ns	*	**	ns	ns
**Anderson-Darling test**					
**A2***	0.5176	1.438	1.493	0.578	0.4129
**P value**	0.1683	0.0008	0.0005	0.1191	0.31
**Passed normality test (alpha=0.05)?**	Yes	No	No	Yes	Yes
**P value summary**	ns	***	***	ns	ns
**Shapiro-Wilk test**					
**W**	0.9339	0.8387	0.8447	0.9251	0.9453
**P value**	0.1481	0.0022	0.0027	0.0969	0.254
**Passed normality test (alpha=0.05)?**	Yes	No	No	Yes	Yes
**P value summary**	ns	**	**	ns	ns
**Kolmogorov-Smirnov test**					
**KS distance**	0.145	0.2831	0.234	0.1611	0.1463
**P value**	>0.1000	<0.0001	0.0028	>0.1000	>0.1000
**Passed normality test (alpha=0.05)?**	Yes	No	No	Yes	Yes
**P value summary**	ns	****	**	ns	ns
Number of values	22	22	22	22	22

Supplemental Table 5: Multiple Linear Regression of cell-associated UTR-deficient RNAs vs IP10 in serum

Variable	β	95% CI (asymptotic)	P value	P value summary
Intercept	0.1235	-0.5675 to 0.8145	0.7108	ns
UTR-deficient RNA	0.4581	0.03540 to 0.8807	0.0353	*
Age @ enrollment	0.007245	-0.006340 to 0.02083	0.2762	ns
ART Years	-0.004207	-0.03632 to 0.02790	0.7855	ns
Sex @ birth	-0.2098	-0.5890 to 0.1695	0.2593	ns

Supplemental Table 6: Multiple Linear Regression of cell-associated UTR-deficient RNAs vs TNFα in serum

Variable	β	95% CI (asymptotic)	P value	P value summary
Intercept	1.167	0.6041 to 1.730	0.0004	***
UTR-deficient RNA	0.4139	0.06950 to 0.7583	0.0213	*
Age @ enrollment	0.002356	-0.008713 to 0.01343	0.659	ns
ART Years	-0.004376	-0.03054 to 0.02179	0.7285	ns
Sex @ birth	0.1226	-0.1864 to 0.4316	0.4142	ns

Supplemental Table 7: Multiple Linear Regression of cell-associated UTR-deficient RNAs vs IL-8 in serum

Variable	β	95% CI (asymptotic)	P value	P value summary
Intercept	-0.1648	-1.190 to 0.8602	0.7386	ns
UTR-deficient RNA	0.6705	0.04350 to 1.297	0.0375	*
Age @ enrollment	0.01006	-0.01009 to 0.03021	0.307	ns
ART Years	0.007806	-0.03982 to 0.05544	0.7338	ns
Sex @ birth	-0.247	-0.8096 to 0.3155	0.3672	ns
